# Elucidating the Role of a Calcium-Binding Loop in an *x*-Prolyl Aminodipeptidase from *Lb. helveticus*

**DOI:** 10.1021/acsomega.3c05639

**Published:** 2023-09-14

**Authors:** Stephanie Ryder, Jacob Pedigo, Deanna Dahlke Ojennus

**Affiliations:** Department of Chemistry, Whitworth University, 300 W. Hawthorne Rd., Spokane, Washington 99251, United States

## Abstract

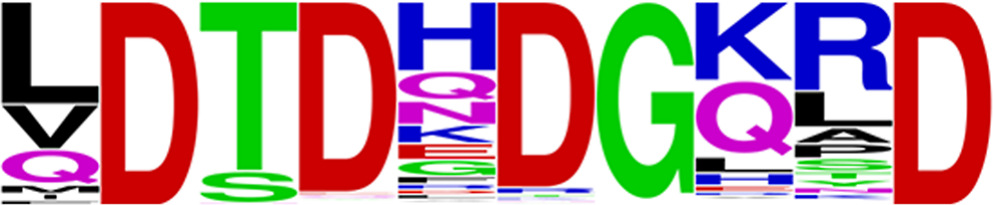

Prolyl aminodipeptidase (PepX) is an α/β hydrolase that cleaves at penultimate N-terminal prolyl peptide bonds. The crystal structure of PepX from *Lactobacillus helveticus* exhibits a calcium-binding loop within the catalytic domain. The calcium-binding sequence of xDxDxDGxxD within this loop is highly conserved in PepX proteins among lactic acid bacteria, but its purpose remains unknown. Enzyme activity is not significantly affected in the presence of the metal chelator ethylenediaminetetraacetic acid (EDTA), nor in the presence of excess calcium ions. To eliminate calcium binding, D196A and D194A/D196A mutations were constructed within the conserved calcium-binding sequence motif. Enzyme activity and stability of the D196A mutant were comparable to the wild-type enzyme by colorimetric kinetic assays and protein thermal shift assays. However, the D194A/D196A mutant was inactive though it retained native-like structure and thermal stability, contradicting the EDTA and calcium titration results. This suggests calcium binding to PepX may be essential for activity.

## Introduction

Calcium-binding proteins (CaBPs) are found throughout prokaryotes, archaea, and eukaryotes. These proteins often function as Ca^2+^ sensors, proteins that transduce or translate calcium ion concentrations into cellular responses often through a conformational change, or as Ca^2+^ buffers which help to modulate calcium ion concentrations within the cell.^1−3^ The role of calcium ions as signals, secondary messengers, and signal transducers is well established in eukaryotes,^4,5^ yet is not fully understood in bacteria. However, CaBPs in prokaryotes have been shown to be involved in processes such as homeostasis, gene expression, chemotaxis, and signaling.^6−8^

**Figure 1:**
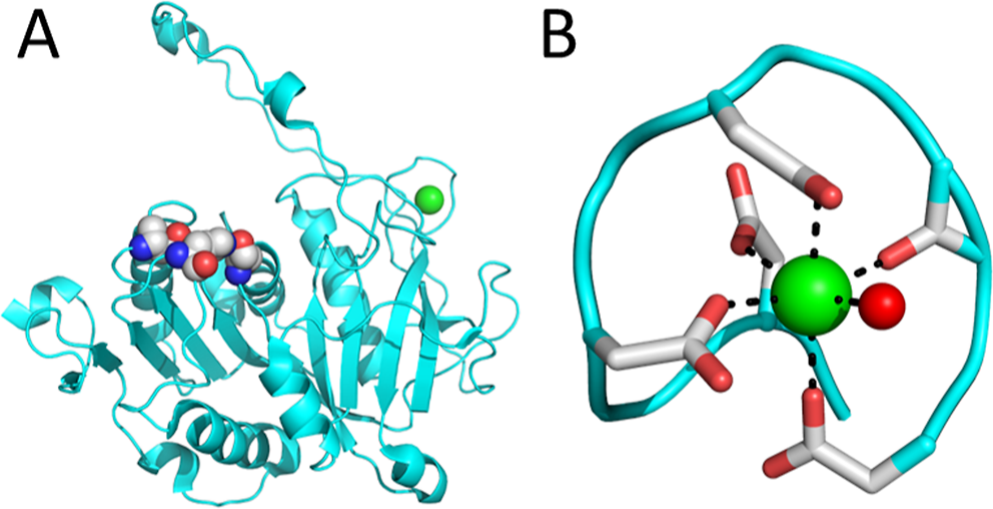
(A) Three-dimensional structure of the α/β hydrolase domain (residues 183–388 and 463–573) of PepX from *Lb. helveticus* shown as a cyan cartoon diagram (PDB 6NFF). Catalytic triad residues S363, D483, and H514 displayed as spheres in CPK coloring. A single bound calcium ion located between β strands 1 and 2 of the hydrolase is shown as a green sphere. (B) Calcium-binding loop (residues 194–202). Atomic radii of the calcium ion (green) and oxygen of HOH1137 (red) set to approximately 50% of actual size for easier visualization of coordinate bonds. Metal coordination bonds to D194, D196, D198, D202, the carbonyl oxygen of K200 and HOH1137 are all between 2.3 and 2.5 Å (black dashed lines).

Prolyl aminodipeptidase (PepX) from *Lactobacillus helveticus* is an intracelluar α/β hydrolase with specificity for penultimate prolyl peptide bonds found at the N-terminal end of peptides.^9−12^ In lactic acid bacteria (LAB), this enzyme is important in the final phases of breaking down environmental nutrients containing high amounts of proline once they enter the cell, such as casein-derived peptides found in milk.^13,14^ When the crystal structure of PepX from *Lb. helveticus* was determined, a calcium ion was found to occupy a loop between the first and second β-strands of the catalytic domain (Figure 1).^15^ The amino acid sequence of this loop contains a calcium-binding motif sequence of DxDxDG typical in calcium-binding loops of EF-hands,^16−18^ as well as other structural contexts,^6,19−21^ in which the three aspartic acid residues within the loop are involved in coordinating the calcium ion. In canonical EF hands, the structural motif is found in a loop between two α-helices and consists of 12 semiconserved residues in positions 1, 3, 5, 7, 9, and 12 which are involved in coordinating the calcium along with a seventh position which is typically occupied by water to form a pentagonal bipyramid.^18,22,23^ The sequence and coordination pattern found in the *Lb. helveticus* PepX calcium-binding site most closely resembles a structural motif proposed by Denesyuk et al. called a calcium-binding blade-like site, in which the DxDxDG sequence is embedded within a 10 amino acid structural motif that forms a closed loop between secondary structural elements and that contains the consensus sequence xDx[DN]xDGxxD (LDTDHDGKSD in the case of *Lb. helveticus* PepX).^24^ Tetragonal bipyramid coordination is achieved by aspartates at positions 2, 4, 6, and 10 which form coordinate bonds while a backbone carbonyl at position 8 also coordinates with the ion, and the sixth position is occupied by either water or an amino acid distant from the loop.

The calcium ion in PepX is located 27 Å away from the nucleophilic serine of the catalytic triad, thus it likely is not involved directly in catalysis. PepX is known to form dimers,^11,25^ but the calcium ion is located 15 Å away from the closest residue at the dimerization interface, thus dimerization would not bring the calcium ion in proximity to the active site of another subunit. These observations raise interesting questions about other possible roles of the calcium-binding motif. In this study, we examined the conservation of the xDx[DN]xDGxxD sequence among PepX enzymes in LAB and tested the activity and thermal stability of the wild-type (w.t.) enzyme compared to the site-directed mutants designed to eliminate calcium binding.

## Results and Discussion

**Figure 2:**
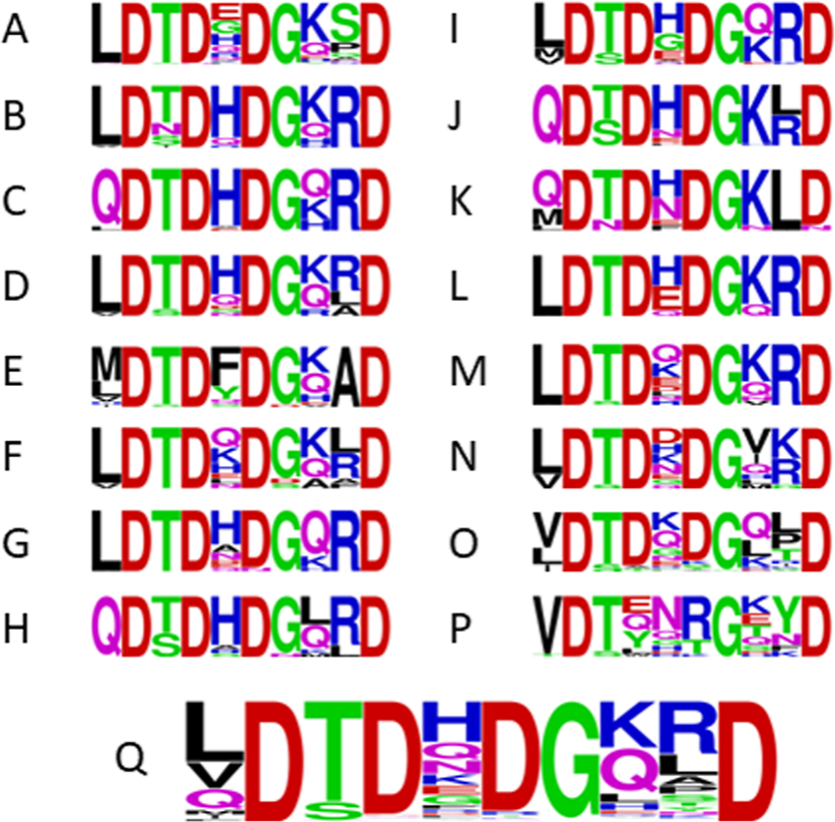
Sequence logo of the frequency of amino acids in the calcium-binding motif of PepX for LAB genera containing at least 10 reference sequences in the order of *Lactobacillales*. (A) *Lactobacillus* (*N* = 42), (B) *Lentilactobacillus* (*N* = 25), (C) *Levilactobacillus* (*N* = 27), (D) *Ligilactobacillus* (*N* = 17), (E) *Limosilactobacillus* (*N* = 23), (F) *Liquorilactobacillus* (*N* = 12), (G) *Lactiplantibacillus* (*N* = 16), (H) *Secundilactobacillus* (*N* = 15), (I) *Lacticaseibacillus* (*N* = 43), (J) *Companilactobacillus* (*N* = 30), (K) *Apilactobacillus* (*N* = 11), (L) *Pediococcus* (*N* = 10), (M) *Enterococcus* (*N* = 13), (N) *Weissella* (*N* = 12), (O) *Streptococcus* (*N* = 95), (P) *Lactococcus* (*N* = 21), (Q) combined results for *Lactobacillales* (*N* = 475).

A homology search for all LAB PepX sequences under the order *Lactobacillales* returned 535 hydrolase reference sequences, 504 of which were identified as Xaa-Pro dipeptidyl-peptidases. These reference sequences for which both a genus and species were identified (475 total) were aligned with the *Lb. helveticus* sequence. The frequency of amino acids in positions 1–10 of the calcium blade-like site are shown in Figure 2 for those genera with at least 10 representative sequences.

The sequence of the calcium-binding blade-like site (xDx[DN]xDGxxD) is highly conserved in PepX proteins among LAB. There is strict conservation in 9 of the 16 genera, including *Lactobacillus*, and the sequence is still highly conserved in the rest except for *Lactococcus* where aspartates in positions 4 and 6 always vary. The aspartate in position 6 of *Streptococcus* PepX sequences is also substituted with either serine, lysine, threonine, alanine, or asparagine in 19% of sequences. The aspartates in the calcium-binding consensus sequence are all directly involved in metal coordination, thus substitution may disrupt calcium binding. Another common substitution found among several genera occurs at position 7 in place of the glycine, which is substituted with either alanine (*Streptococcus*), asparagine (*Secundilactobacillus*), aspartate (*Streptococcus*, *Limosilactobacillus*, *Liquorilactobacillus*), cysteine (*Streptococcus*), or serine (*Streptococcus*, *Liquorilactobacillus*). The sterically unhindered glycine is likely necessary to form the tight loop structure and the observed substitutions are all small amino acids.

There are currently only two structures known for bacterial PepX enzymes, a crystal structure by Rigolet et al. of the PepX from *Lactococcus lactis*(25) and the crystal structure of PepX from *Lactobacillus helveticus*.^15^ The calcium-binding motif is not conserved in the *Lactococcus* genus and the *Lc. lactis* sequence is missing two of the important aspartates for metal coordination at positions 4 and 6 (VDTEQKGKND). Thus, it is no surprise that calcium was not observed in the *Lc. lactis* PepX structure like it did for *Lb. helveticus*.

The high degree of conservation of the calcium-binding sequence among all LAB suggests that calcium-binding by PepX is of some significance to the protein’s function or structure. Previously, Stressler et al. tested the activity of *Lb. helveticus* PepX with the addition of ethylenediaminetetraacetic acid (EDTA) or calcium.^26^ EDTA concentrations from 0.1 to 10 mM appeared to have no inhibitory effect on the activity of the enzyme, and if anything, slightly increased the activity. Similarly, calcium titrations from 0.1 to 10 mM had a negligible effect at lower concentrations, but decreased activity to 59% at 10 mM. However, the calcium titrations were performed in phosphate buffer, likely leading to calcium phosphate precipitates. Stressler et al. also observed that other divalent metals inhibited the enzyme, especially copper and mercury.

**Figure 3:**
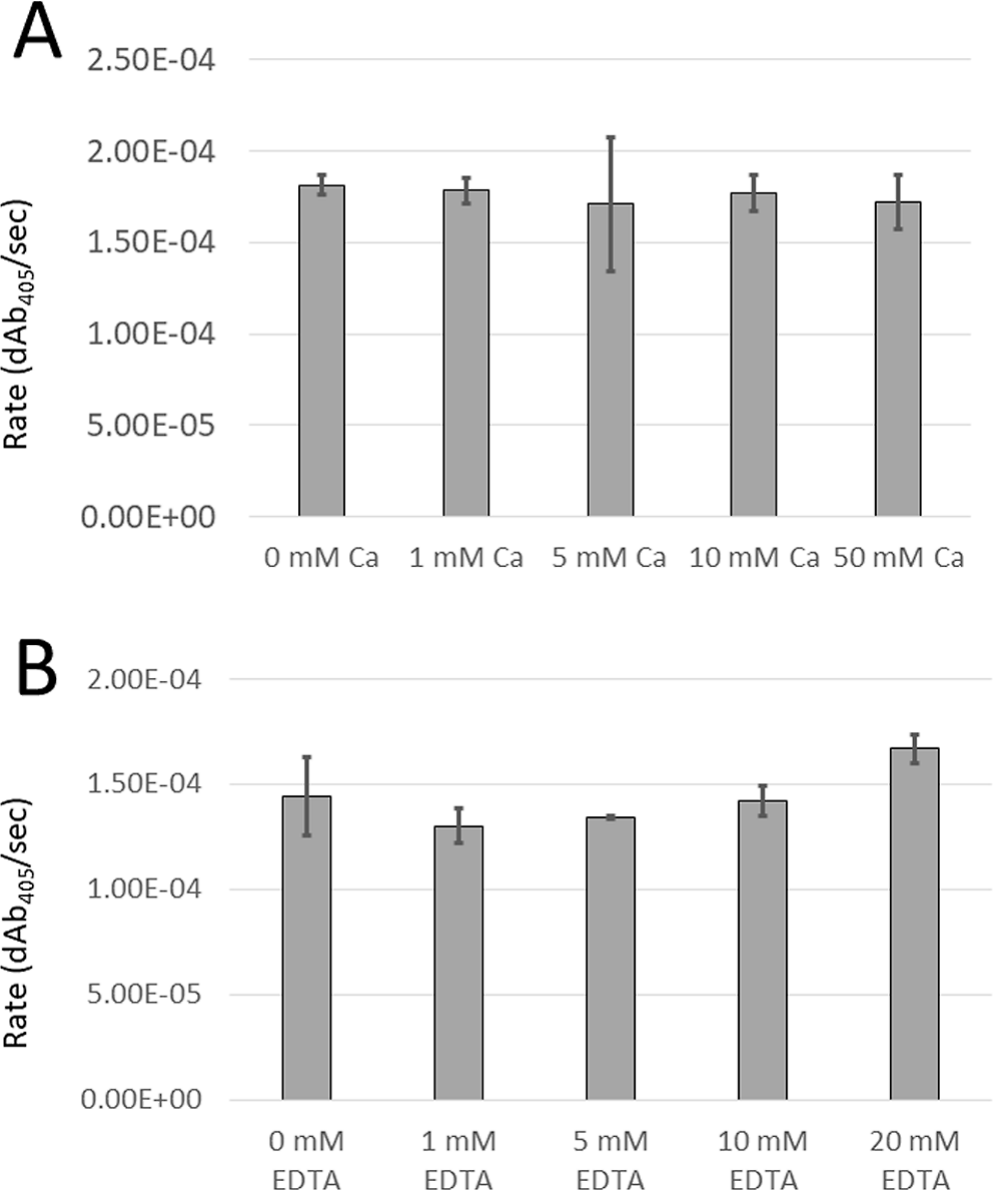
Rate of PepX catalysis measured as a change in absorbance at 405 nm/s for a 10-min assay using the substrate mimic GPpNA. Reactions carried out at 37 °C in 50 mM MES, pH 6.5, 10 mM NaCl, 250 μM GPpNA, and 1 nM PepX. Error bars represent standard deviation of triplicate runs. (A) EDTA concentration fixed at 5 mM with calcium chloride titrated from 0 to 50 mM. (B) Calcium chloride concentration fixed at 5 mM with EDTA titrated from 0–20 mM.

To confirm the observations made by Stressler et al., the PepX enzyme was titrated with EDTA and Ca^2+^ and tested for activity. EDTA was held constant at 5 mM and calcium chloride was titrated from 0 to 50 mM in a 2-(*N*-morpholino) ethanesulfonic acid (MES) buffer to prevent calcium precipitates. Additionally, calcium was held constant at 5 mM and EDTA was titrated from 0 to 20 mM (Figure 3). Neither EDTA nor calcium ions had any significant impact on the activity of PepX (0–50 mM Ca^2+^*p* = 0.37, 0–20 mM EDTA *p* = 0.14). If the calcium-binding site regulates enzyme activity, we may predict that the addition of EDTA would increase or decrease activity. However, that is assuming the EDTA is sufficient to strip any bound calcium from the enzyme. Instead, the site may have a very high affinity for calcium and EDTA may be insufficient to remove it.

**Figure 4:**
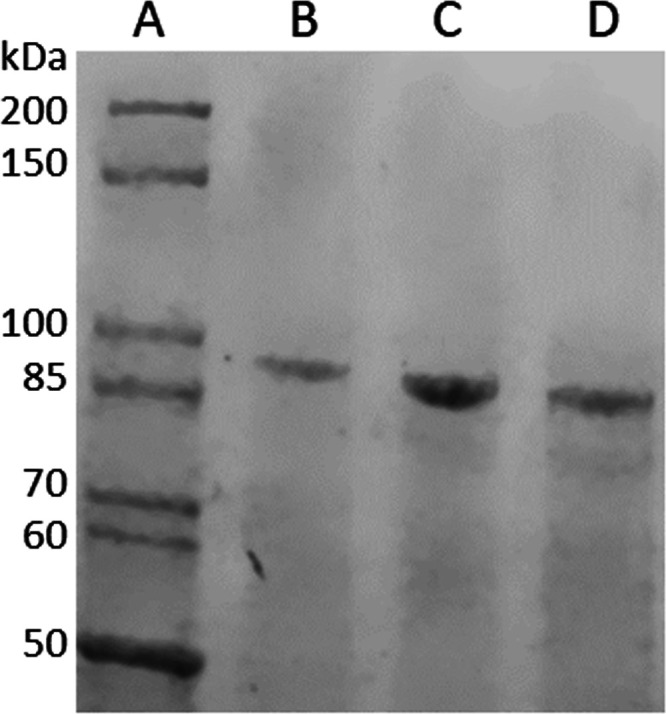
Tris-glycine 4–15% gradient SDS-PAGE of approximately 200 ng of purified PepX proteins. (A) Unstained broad range protein standard (NEB P7717S), (B) w.t. PepX, (C) D196A PepX, and (D) D194A/D196A PepX. The recombinant PepX protein has a molecular weight of 92.5 kDa. Image obtained by coomassie fluorescence using the 700 nm channel of an LI-COR Odyssey Fc imager.

To establish whether calcium-binding has an effect on the activity, two site-directed mutants were constructed in which the aspartate at position 4 of the xDx[DN]xDGxxD calcium-binding blade-like sequence was mutated to alanine (D196A) as well as at positions 2 and 4 (D194A/D196A). D196A and D194A/D196A behaved the same as w.t. PepX during expression and purification (Figure 4).

**Figure 5:**
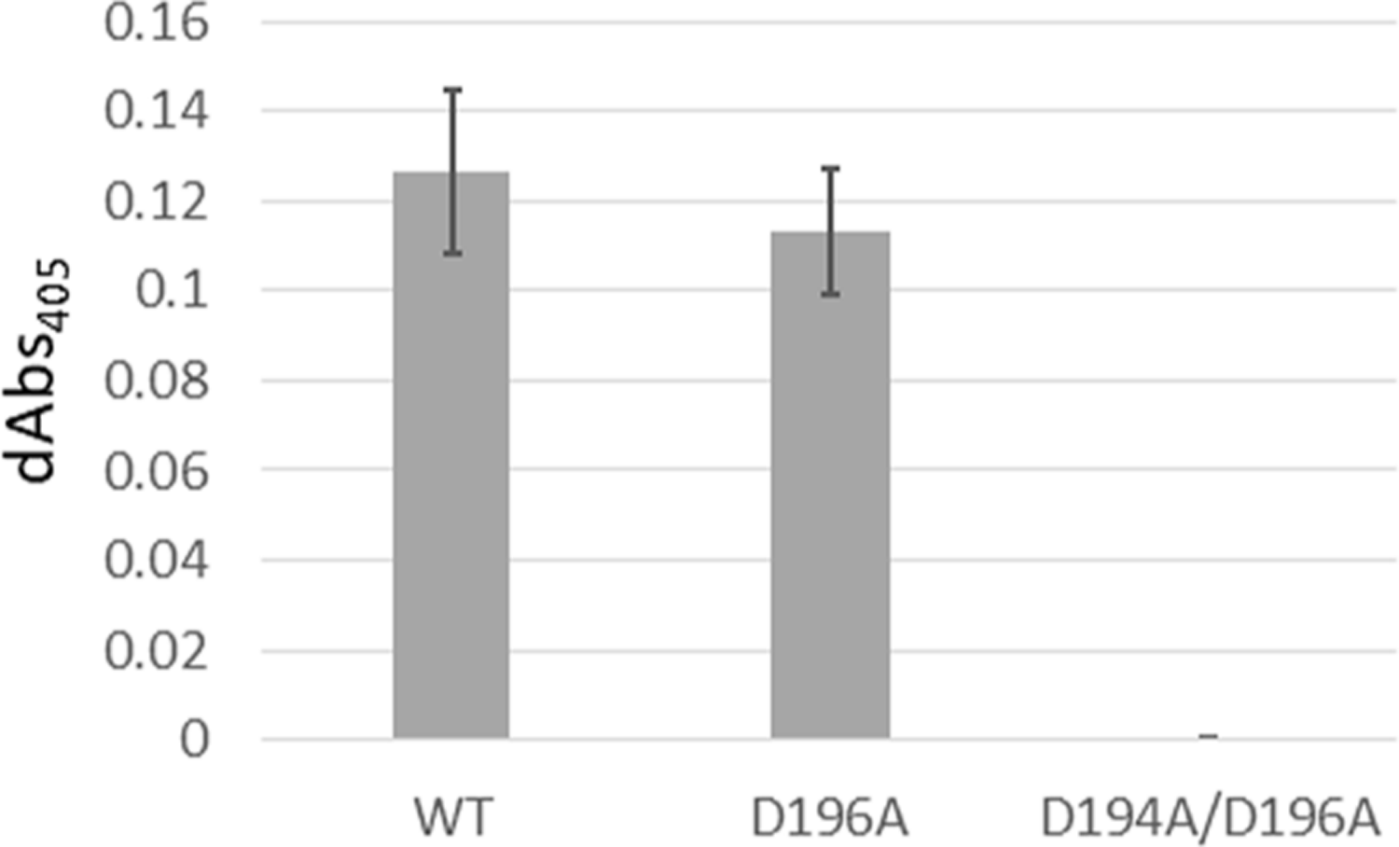
Change in absorbance at 405 nm for a 5-min activity assay of either 200 nM w.t. PepX, D196A, or D194A/D196A in 50 mM MES, pH 6.5 and 200 μM GPpNA at 40 °C. Error bars represent standard deviation of triplicate runs.

The activity of w.t. PepX, D196A, and the D194A/D196A mutants were compared in a 5-min assay (Figure 5). Although the average product formed for the D196A mutant was slightly less than w.t. PepX, the difference was not statistically significant (*p* = 0.36). However, the D194A/D196A double mutant was inactive. The temperature of 40 °C was selected for the activity assays to match conditions previously reported for kinetic data of PepX as it is the optimal temperature for the w.t. enzyme;^15,27^ however, the D194A/D196A mutant was also determined to be inactive both at room temperature and in 50 mM potassium phosphate buffer pH 6.5 while w.t. PepX and D196A always displayed comparable activity (data not shown).

**Figure 6:**
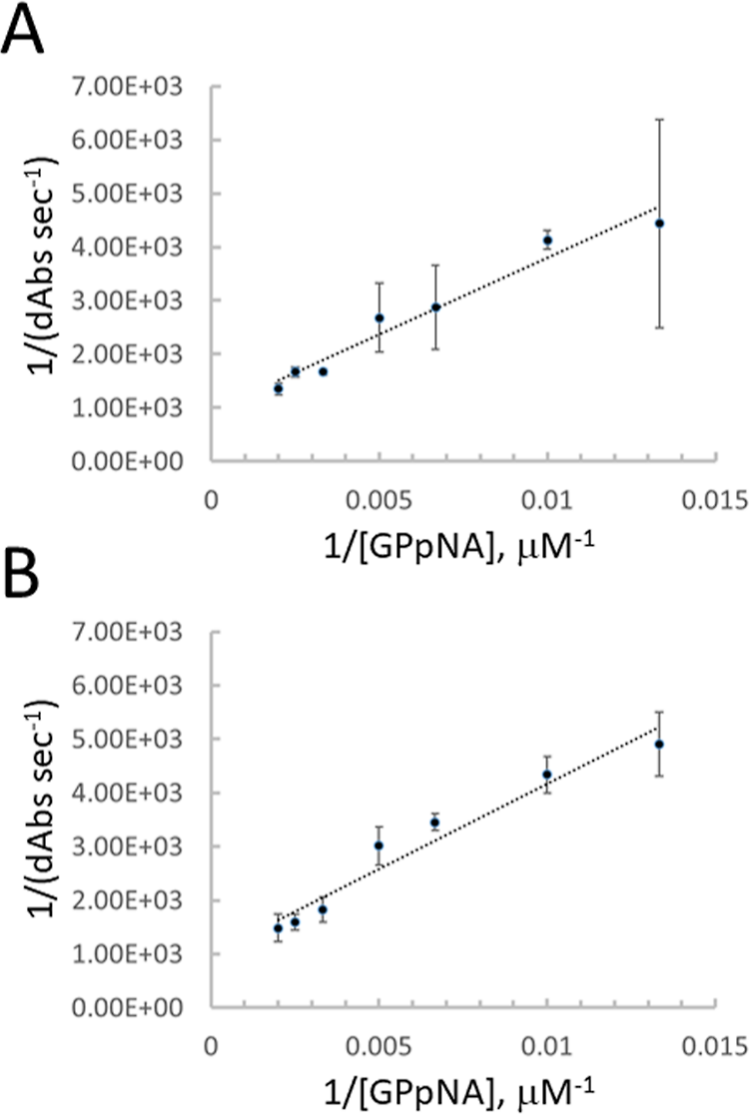
Lineweaver–Burk plots of (A) w.t. PepX and (B) D196A. Initial rates were measured at 40 °C with 75, 100, 150, 200, 300, 400, and 500 μM GPpNA and 200 nM enzyme in 50 mM MES pH 6.5. Error bars represent standard deviation of triplicate runs. The *x*-intercepts of each plot provided a *K*_m_ for w.t. PepX of 302 and 315 μM for D196A.

A full kinetic characterization of w.t. PepX and the D196A mutant was done by a Lineweaver–Burk analysis (Figure 6). The Michaelis–Menten constant (*K*_m_) of w.t. PepX was determined to be 302 μM based on the *x*-intercept of the plot while the *K*_m_ of the D196A mutant was 315 μM. Both are reasonably close to the previously reported range of *K*_m_ values for w.t. PepX against a GPpNA substrate of 250–310 μM.^15,27^ A nonlinear least-squares regression of the data fit to the Michaelis–Menten equation confirmed that the *K*_m_ values, although slightly different, were within error of one another (data not shown). Thus, the single mutation to the calcium-binding sequence has negligible impact on the activity of PepX but the double mutation eliminates all activity.

**Figure 7:**
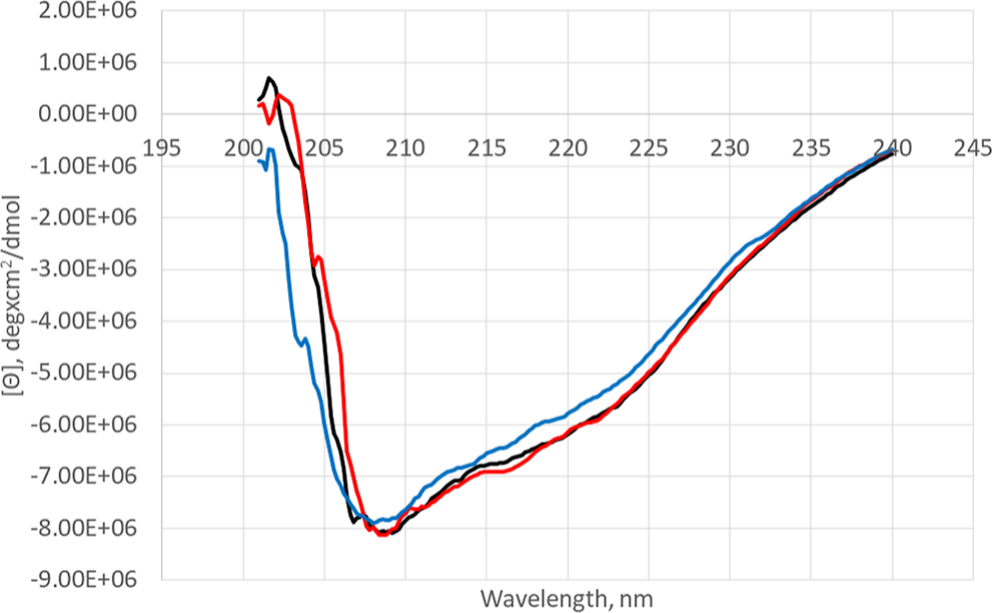
Far-UV CD spectrum showing molar ellipticity for w.t. PepX (black), D196A single mutant (blue), and the D194A/D196A double mutant (red) in 50 mM potassium phosphate pH 6.5.

Loss of activity in the D194A/D196A construct may be due to loss of calcium binding, or it may be due to misfolding of the protein. To evaluate the potential effects on the global structure of the protein by each mutation, mutants were examined by far-UV circular dichroism (CD) spectropolarimetry and compared to w.t. PepX (Figure 7). All three spectra exhibit characteristics of a folded protein with expected local minima at 208, 218, and 222 nm for a protein containing both α-helix and β-sheet structures. The similarity in CD spectra suggests that the double mutation does not significantly change the global structure of PepX. However, a small conformational change may still lead to inactivation.

**Figure 8:**
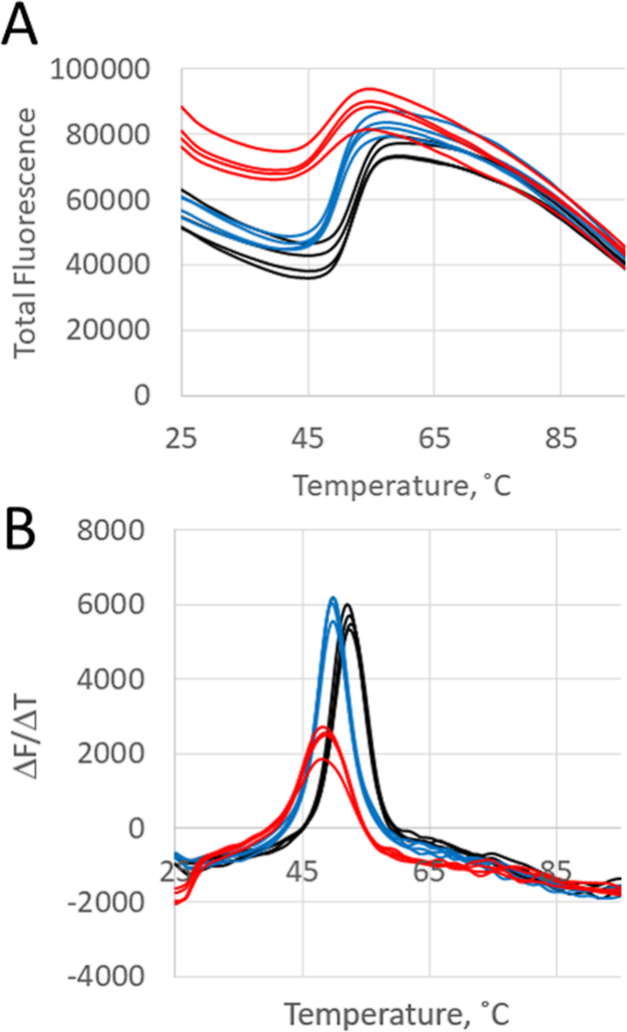
Protein thermal shift assay of w.t. PepX (black), D196A single mutant (blue), and D194A/D196A double mutant (red). Assays done in 50 mM potassium phosphate pH 6.5, 10 mM sodium chloride and 0.25 mg/mL of each enzyme. (A) Measure of total fluorescence vs temperature and (B) first-derivative plot showing change in fluorescence (Δ*F*) with change in temperature (Δ*T*) vs temperature. Inflection points for four trials of each enzyme were averaged to give a *T*_m_ of 52.4 ± 0.2 °C (S.D.) for w.t. PepX, 49.8 ± 0.1 °C (S.D.) for D196A, and 48.5 ± 0.3 °C (S.D.) for D194A/D196A. Differences between averages were statistically significant with *p* ≤ 0.001 for the w.t. PepX average compared to each mutant as well as D196A compared to D194A/D196A.

The relative thermal stability of w.t. PepX was compared to the D196A and D194A/D196A mutants by protein thermal shift assay (Figure 8). Mutations in the calcium-binding loop have a small, but measurable effect on the protein stability. The w.t. PepX had an average melting point approximately 2 degrees higher than the single mutant and approximately 3 degrees higher than the double mutant. However, the relative thermal stability difference between D194A/D196A and D196A is so small, it does not immediately suggest a reason why the double mutant is inactive while the single mutant retains activity.

It is interesting to note that the starting value for total fluorescence in the protein thermal shift assays is much higher for D194A/D196A than either D196A or w.t. PepX and the transition from folded to unfolded is a slightly broader transition. Since fluorescence increases as the thermofluor interacts with exposed hydrophobic patches on the protein, the difference between the starting fluorescence observed for the double mutant when compared to the single mutant or w.t. PepX suggests there is a structural difference between the inactive and active forms of the enzyme that was not immediately apparent in the CD spectra. D194A/D196A could be structurally distinct from w.t. PepX and therefore it interacts with the dye differently. Or there could be conformational heterogeneity in the protein sample, possibly due to the presence of partially unfolded or degraded protein, although that was not indicated in the CD spectrum. Another possibility is that the conformational dynamics could be altered in the double mutant to allow the thermofluor to interact with internal areas of the protein more readily. Any of these could potentially explain the inactivity of the D194A/D196A PepX construct as well as the higher starting fluorescence in the thermofluor assays.

An interesting example that closely resembles the observations made here for PepX is a thermophillic endocellulase (EGPf) from the archaeon *Pyrococcus furiosus* which adopts a jelly-roll structure.^28^ The coordinating groups in the cellulase also form a tetragonal bipyramid and are located in a calcium-binding loop between two β-strands containing an xDxDxDGxxE sequence where the fifth coordinating position is a glutamate instead of an aspartate and the sixth coordinating position is a distant aspartate located in an extended loop, rather than water. The calcium-binding site is also located 27 Å away from the active site. When activity of the enzyme was tested in the presence of EDTA, there was no change in cellulase activity. However, differential scanning calorimetry (DSC) measurements indicated calcium binding contributed at least 5 °C toward thermal stability of the enzyme.

The strict conservation of the calcium-binding sequence in PepX among most genera of LAB suggests a necessary structural or regulatory role for calcium beyond a modest thermal stabilization. There are many examples among prokaryotic proteins where calcium-binding loops play critical roles in stabilizing structures. For example, in the subtilisin superfamily, bound calcium ions have structural roles that lead to proper folding and resistance to autolysis in addition to improving thermostability.^29−32^ But there are fewer prokaryotic examples where calcium regulates the enzyme activity. CaBPs with the linear Dx[D/N]xDG motif appear in many different structural contexts other than the canonical helix–loop–helix EF-hand structure and are mostly believed to have structural or stabilizing roles in prokaryotes rather than regulatory roles as predominantly observed in eukaryotes.^19,20^ Some exceptions include the pilus biogenesis factor PilY1 from *Pseudomonas aeruginosa* where calcium regulates motility,^33^ the *Sphingomonas* periplasmic alginate-binding protein which may act as a sensor,^19,34^ and a d-glucose/d-galactose-binding protein from *Escherichia coli* in which calcium not only stabilizes the structure but also can be proposed to regulate large-scale conformational dynamics between open and closed states.^35,36^

Calcium may simply be needed by PepX for constitutive activity. However, it is not unreasonable to assume PepX activity could be regulated by cytosolic calcium ion concentrations, whether through a structural change or an alteration in conformational dynamics. LAB are a natural milk microflora and extensively used in making milk fermentation products.^37,38^ Milk, which contains proline-rich casein proteins, is also an environment rich in calcium,^39^ but bacteria are known to maintain calcium ion homeostasis at levels much lower than the extracellular environment.^6,40−42^*E. coli* have been shown to maintain concentrations of calcium ions in the nanomolar range.^40^ Thus, an enzyme with high affinity for calcium could be regulated under the slow fluctuations of intracellular calcium which have been observed in bacteria,^42^ and which may be affected by external environment.

## Conclusion

The data presented here suggest that the calcium-binding loop of *Lb. helveticus* PepX is potentially an allosteric regulatory site for activity. Further investigation is needed to determine whether calcium binding to PepX merely stabilizes the active form of the enzyme or causes a more significant conformational change between the active and inactive forms. Determination of a dissociation constant would also help in understanding if enzyme activity can be modulated at physiologically relevant calcium ion concentrations. This work adds to a growing body of evidence of calcium signaling processes in prokaryotes.

## Methods

### Sequence Analysis

The sequence of *Lb. helveticus* PepX (UniProt Q59485) was analyzed by the blastp algorithm^43,44^ on the NCBI server with an Expect threshold of 0.05. The search was restricted to the RefSeq Select proteins database for all reference sequences found under the order *Lactobacillales* (taxid: 186826). Amino acid frequency plots were prepared from aligned calcium-binding sequences using WebLogo.^45^

### Construction of PepX Mutants

Construction of a recombinant *Lb. helveticus* PepX gene with an N-terminal six-histidine tag in a pET14b expression vector was described previously (NCBI 6NFF_A).^15^ A single PepX mutation of D196A and a double mutation of D194A and D196A were prepared using a Q5 Site-Directed Mutagenesis Kit (New England Bio-Labs). Custom mutagenic primers were purchased from Life Technologies Corp. (Thermo Fisher Scientific) with these sequences: 5′-ATGAAATATAACCAATATGCTTACG-3′ and 5′-ACTGAAATCAAGTTTTATGAAAAATAA-3′ for the D196A mutation and 5′-CATGATGGCAAGAGTGATTTAATTCAAGTTAC-3′ and 5′-GGCAGTGGCAAGATCAGTTTCGACATAAAC-3′ for D194A/D196A mutations. Mutations were confirmed by DNA sequencing (Genewiz).

### Expression and Purification of PepX, D196A, and D194A/D196A

Both the w.t. and the PepX mutants were expressed and purified according to a previously described method.^15^ Briefly, protein expression was induced with isopropyl β-d-1-thiogalactopyranoside (IPTG) in BL21(de3)pLysS cells and then purified from cell lysates by ammonium sulfate precipitation, nickel affinity chromatography, and anion exchange chromatography. Final purified protein was dialyzed into either 50 mM MES pH 6.5 or 50 mM potassium phosphate buffer pH 6.5. Protein concentrations were determined by Bradford assay.^46^

### Enzyme Activity Assays

All activity assays were performed with the substrate mimic Gly-Pro-*p*-nitroanilide hydrochloride (G0513 Sigma-Aldrich) and monitored at 405 nm with an ELx808 Absorbance Microplate Reader (Bio-Tek). Calcium chloride and EDTA titrations with w.t. PepX were performed in triplicate at 37 °C in 50 mM MES, pH 6.5, 10 mM NaCl, 250 μM GPpNA, and 1 nM PepX. Calcium chloride concentrations were varied from 0 to 50 mM at a fixed concentration of 5 mM EDTA. Conversely, EDTA concentrations were also varied from 0 to 20 mM at a fixed concentration of 5 mM calcium chloride. The change in absorbance was monitored for 10 min with a reading every 15 s. Rates were calculated from the slope as the change in absorbance at 405 nm per second. The full kinetic characterization of w.t. and D196A PepX was processed in triplicate with varying concentrations of GPpNA (75, 100, 150, 200, 300, 400, 500 μM) in 50 mM MES, pH 6.5 and 200 nM enzyme at 40 °C. Change in absorbance at 405 nm was measured at 15 s intervals for 400 s. Initial reaction rates were determined by the slope of the best-fit line as the change in absorbance at 405 nm per s for the linear part of the curve during the first 6 min assayed. Data were analyzed using a Lineweaver–Burk plot and the *K*_m_ was determined from the *x*-intercept (−1/*K*_m_) of the best-fit line. The total change in absorbance at 405 nm was also measured for a 5 min reaction with 200 μM GPpNA substrate. All averages were analyzed by two-tailed *t*-test assuming unequal variances.

### CD Spectropolarimetry

The CD of proteins was analyzed using a JASCO J-815 spectrometer. Scans from 201 to 240 nm were obtained with a bandwidth of 1 nm, data pitch of 0.2 nm, and scan rate of 100 nm/min using a 2 mm cuvette at 25 °C. Protein samples ranged from 0.25 to 0.41 mg/mL in 50 mM potassium phosphate buffer, pH 6.5. Ten scans were averaged for each sample and measurements of ellipticity in millidegrees were converted to molar ellipticity (deg × cm^2^/dmol) based on the concentration of each sample.

### Protein Thermal Shift Assays

Relative stability of w.t. PepX compared to the D196A and D194A/D196A mutants was measured by protein thermal shift assay using a Thermo Fisher Protein Thermal Shift Dye Kit (Cat. #4461146) and an Applied Biosystems StepOne Real-Time PCR system quantitative thermocycler. The enzymes were prepared at a final concentration of 0.25 mg/mL in 50 mM potassium phosphate pH 6.5, 10 mM sodium chloride with protein thermal shift dye according to the manufacturer’s instructions. Thermal melts were performed in quadruplicate. The first derivative of the change in fluorescence with change in temperature was used to find the melting temperature (*T*_m_) using the Protein Thermal Shift Software (Version 1.4) from Applied Biosystems. Average *T*_m_s were analyzed by a two-tailed *t*-test assuming unequal variances.

## Abbreviations

CaBP: calcium-binding protein
CD: circular dichroism
CPK: Corey–Pauling–Koltun
DSC: differential scanning calorimetry
EDTA: ethylenediaminetetraacetic acid
GPpNA: Gly-Pro-*p*-nitroanilide
IPTG: isopropyl β-d-1-thiogalactopyranoside
LAB: lactic acid bacteria
MES: 2-(*N*-morpholino) ethanesulfonic acid
NCBI: National Center for Biotechnology Information
PepX: *x*-prolyl aminodipeptidase
UV: ultraviolet
w.t.: wild-type
